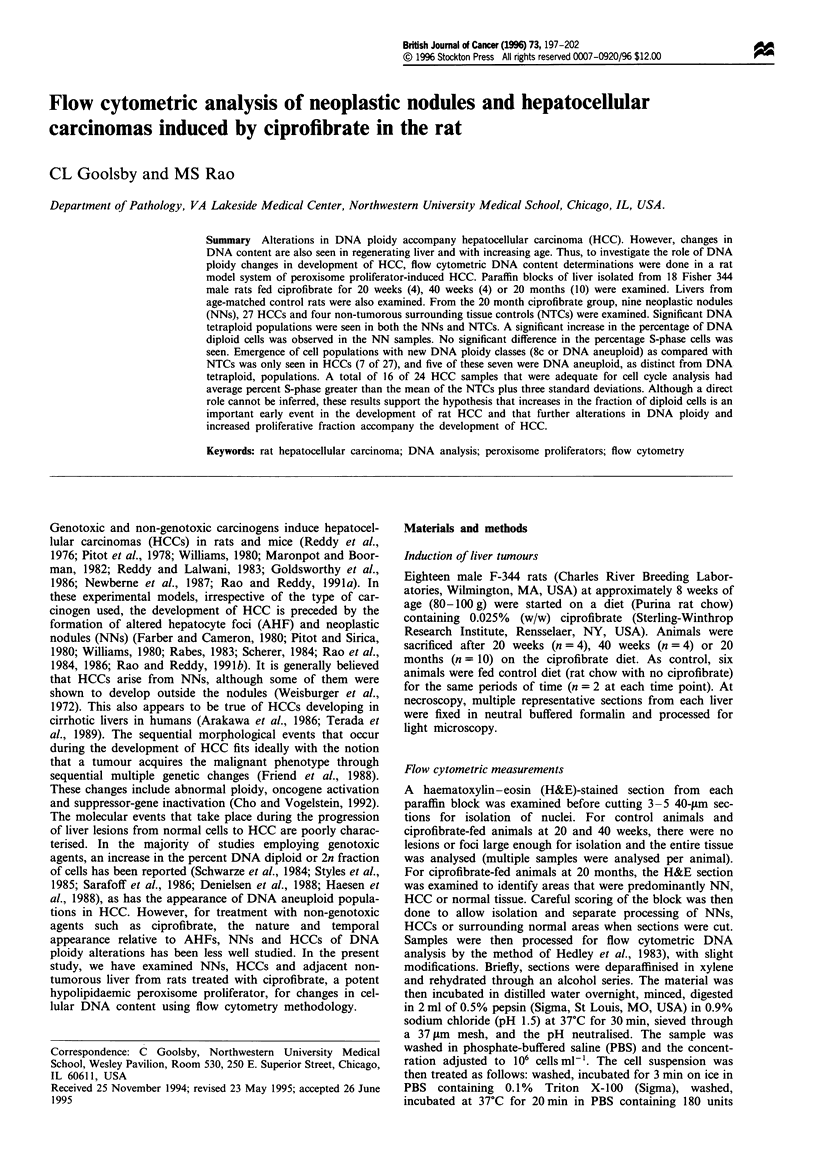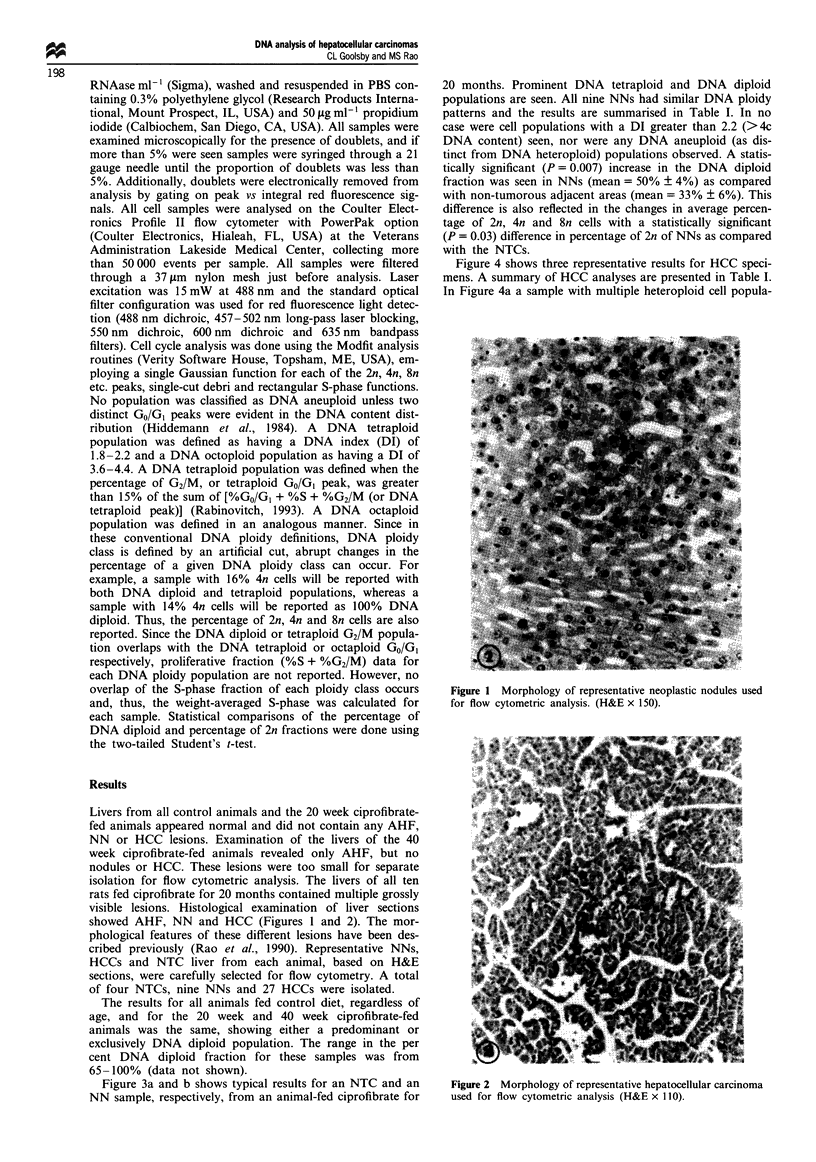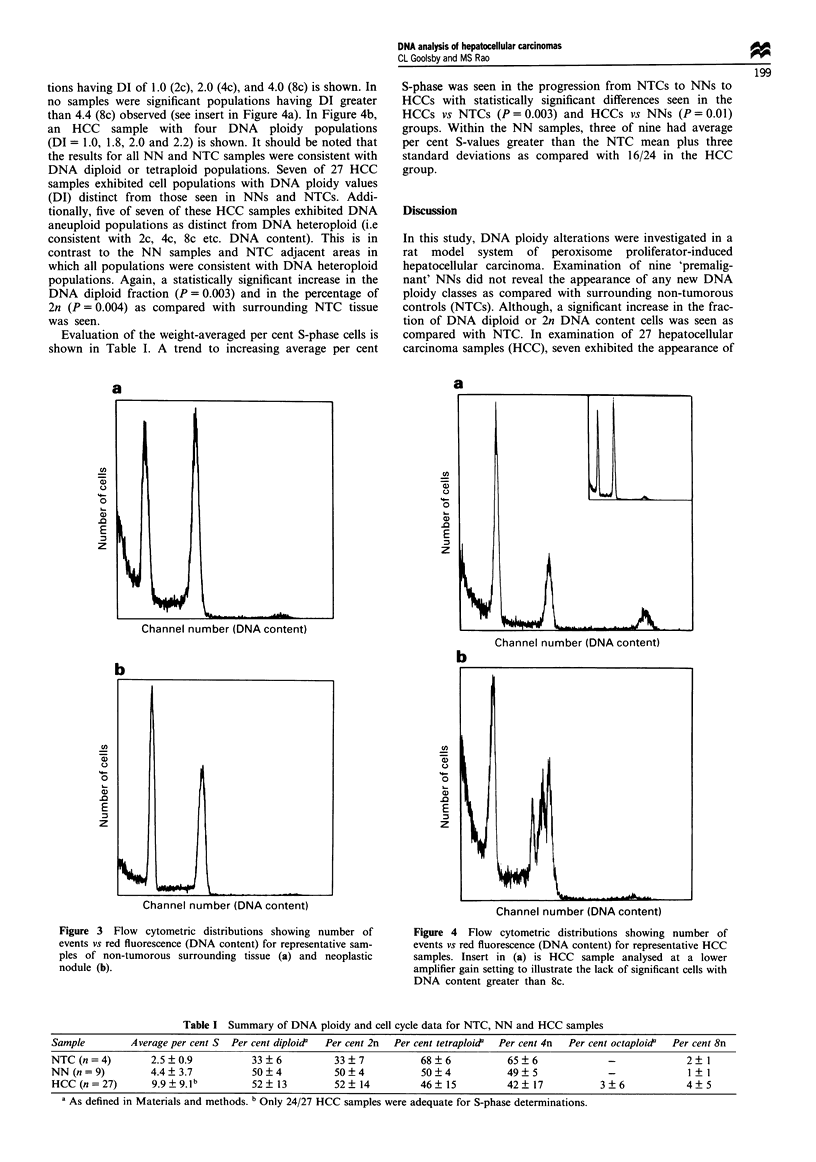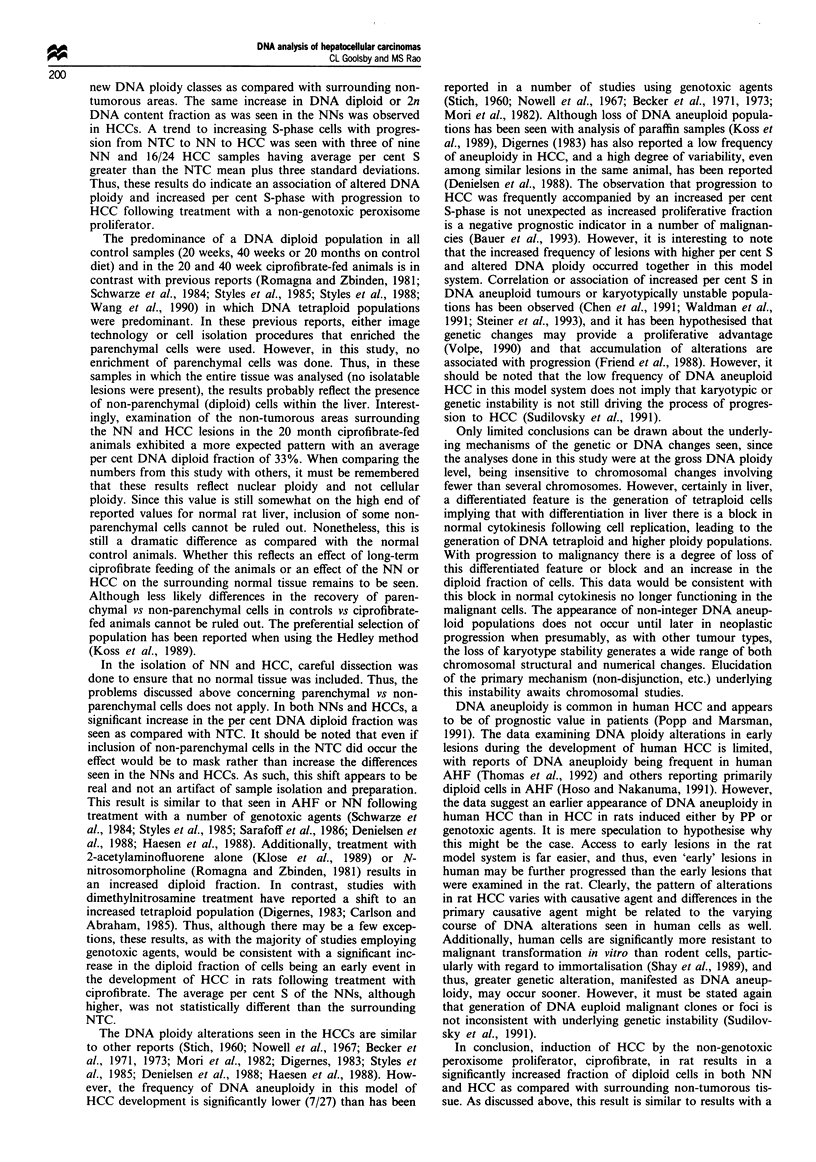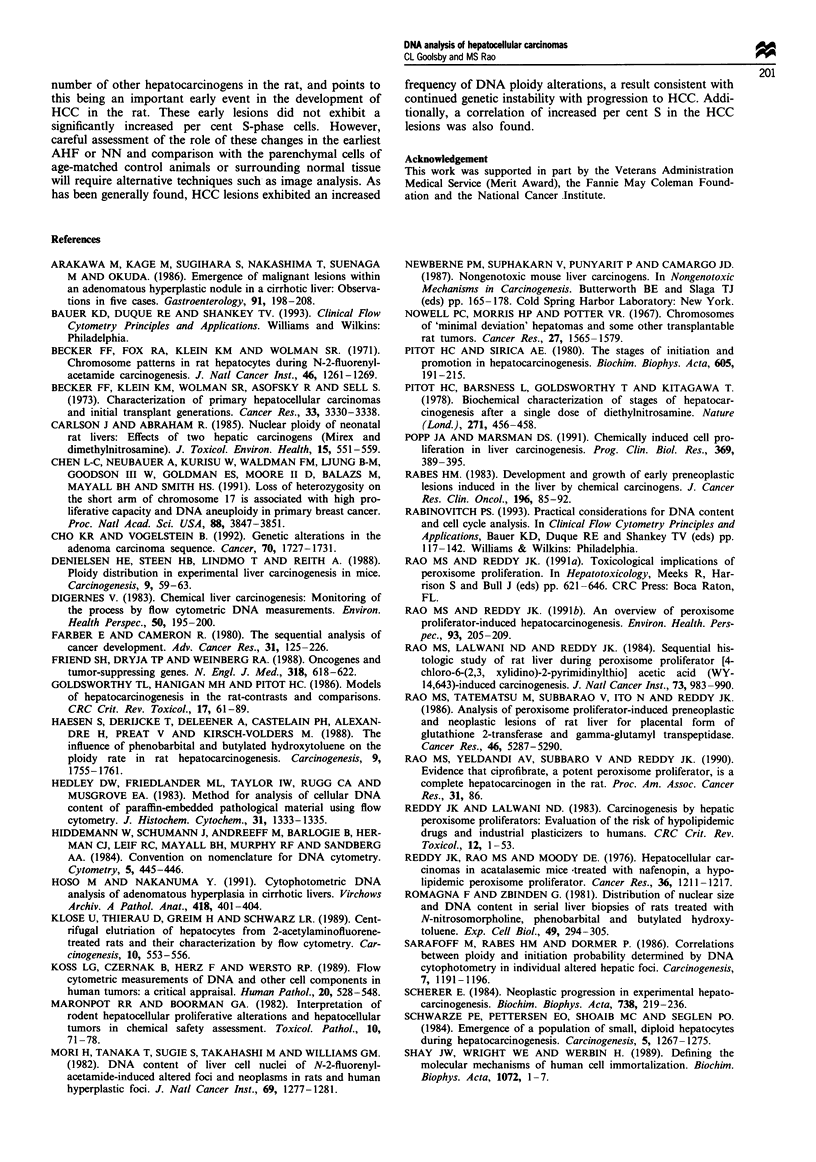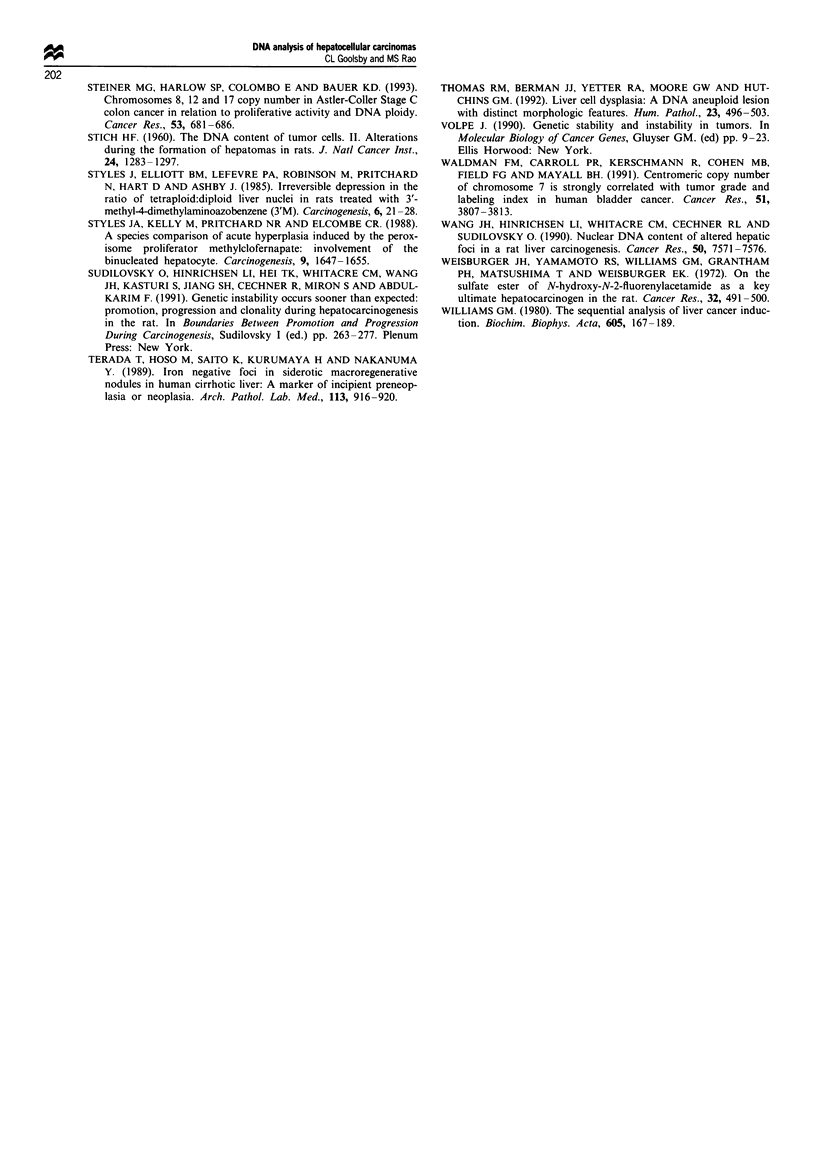# Flow cytometric analysis of neoplastic nodules and hepatocellular carcinomas induced by ciprofibrate in the rat.

**DOI:** 10.1038/bjc.1996.35

**Published:** 1996-01

**Authors:** C. L. Goolsby, M. S. Rao

**Affiliations:** Department of Pathology, VA Lakeside Medical Center, Chicago, IL, USA.

## Abstract

**Images:**


British Journal of Cancer (1996) 73, 197–202
© 1996 Stockton Press All rights reserved 0007–0920/96 $12.00

### Flow cytometric analysis of neoplastic nodules and hepatocellular carcinomas induced by ciprofibrate in the rat

CL Goolsby and MS Rao

*Department of Pathology, VA Lakeside Medical Center, Northwestern University Medical School, Chicago, IL, USA.*

**Summary** Alterations in DNA ploidy accompany hepatocellular carcinoma (HCC). However, changes in DNA content are also seen in regenerating liver and with increasing age. Thus, to investigate the role of DNA ploidy changes in development of HCC, flow cytometric DNA content determinations were done in a rat model system of peroxisome proliferator-induced HCC. Paraffin blocks of liver isolated from 18 Fisher 344 male rats fed ciprofibrate for 20 weeks (4), 40 weeks (4) or 20 months (10) were examined. Livers from age-matched control rats were also examined. From the 20 month ciprofibrate group, nine neoplastic nodules (NNs), 27 HCCs and four non-tumorous surrounding tissue controls (NTCs) were examined. Significant DNA tetraploid populations were seen in both the NNs and NTCs. A significant increase in the percentage of DNA diploid cells was observed in the NN samples. No significant difference in the percentage S-phase cells was seen. Emergence of cell populations with new DNA ploidy classes (8c or DNA aneuploid) as compared with NTCs was only seen in HCCs (7 of 27), and five of these seven were DNA aneuploid, as distinct from DNA tetraploid, populations. A total of 16 of 24 HCC samples that were adequate for cell cycle analysis had average percent S-phase greater than the mean of the NTCs plus three standard deviations. Although a direct role cannot be inferred, these results support the hypothesis that increases in the fraction of diploid cells is an important early event in the development of rat HCC and that further alterations in DNA ploidy and increased proliferative fraction accompany the development of HCC.

**Keywords:** rat hepatocellular carcinoma; DNA analysis; peroxisome proliferators; flow cytometry

Genotoxic and non-genotoxic carcinogens induce hepatocellular carcinomas (HCCs) in rats and mice (Reddy *et al.*, 1976; Pitot *et al.*, 1978; Williams, 1980; Maronpot and Boorman, 1982; Reddy and Lalwani, 1983; Goldsworthy *et al.*, 1986; Newberne *et al.*, 1987; Rao and Reddy, 1991*a*). In these experimental models, irrespective of the type of carcinogen used, the development of HCC is preceded by the formation of altered hepatocyte foci (AHF) and neoplastic nodules (NNs) (Farber and Cameron, 1980; Pitot and Sirica, 1980; Williams, 1980; Rabes, 1983; Scherer, 1984; Rao *et al.*, 1984, 1986; Rao and Reddy, 1991*b*). It is generally believed that HCCs arise from NNs, although some of them were shown to develop outside the nodules (Weisburger *et al.*, 1972). This also appears to be true of HCCs developing in cirrhotic livers in humans (Arakawa *et al.*, 1986; Terada *et al.*, 1989). The sequential morphological events that occur during the development of HCC fits ideally with the notion that a tumour acquires the malignant phenotype through sequential multiple genetic changes (Friend *et al.*, 1988). These changes include abnormal ploidy, oncogene activation and suppressor-gene inactivation (Cho and Vogelstein, 1992). The molecular events that take place during the progression of liver lesions from normal cells to HCC are poorly characterised. In the majority of studies employing genotoxic agents, an increase in the percent DNA diploid or $2n$ fraction of cells has been reported (Schwarze *et al.*, 1984; Styles *et al.*, 1985; Sarafoff *et al.*, 1986; Denielsen *et al.*, 1988; Haesen *et al.*, 1988), as has the appearance of DNA aneuploid populations in HCC. However, for treatment with non-genotoxic agents such as ciprofibrate, the nature and temporal appearance relative to AHFs, NNs and HCCs of DNA ploidy alterations has been less well studied. In the present study, we have examined NNs, HCCs and adjacent non-tumorous liver from rats treated with ciprofibrate, a potent hypolipidaemic peroxisome proliferator, for changes in cellular DNA content using flow cytometry methodology.

Correspondence: C Goolsby, Northwestern University Medical School, Wesley Pavilion, Room 530, 250 E. Superior Street, Chicago, IL 60611, USA
Received 25 November 1994; revised 23 May 1995; accepted 26 June 1995

### Materials and methods

### *Induction of liver tumours*

Eighteen male F-344 rats (Charles River Breeding Laboratories, Wilmington, MA, USA) at approximately 8 weeks of age (80–100 g) were started on a diet (Purina rat chow) containing 0.025% (w/w) ciprofibrate (Sterling-Winthrop Research Institute, Rensselaer, NY, USA). Animals were sacrificed after 20 weeks ($n = 4$), 40 weeks ($n = 4$) or 20 months ($n = 10$) on the ciprofibrate diet. As control, six animals were fed control diet (rat chow with no ciprofibrate) for the same periods of time ($n = 2$ at each time point). At necroscopy, multiple representative sections from each liver were fixed in neutral buffered formalin and processed for light microscopy.

### *Flow cytometric measurements*

A haematoxylin–eosin (H&E)-stained section from each paraffin block was examined before cutting 3–5 40-μm sections for isolation of nuclei. For control animals and ciprofibrate-fed animals at 20 and 40 weeks, there were no lesions or foci large enough for isolation and the entire tissue was analysed (multiple samples were analysed per animal). For ciprofibrate-fed animals at 20 months, the H&E section was examined to identify areas that were predominantly NN, HCC or normal tissue. Careful scoring of the block was then done to allow isolation and separate processing of NNs, HCCs or surrounding normal areas when sections were cut. Samples were then processed for flow cytometric DNA analysis by the method of Hedley *et al.*, 1983), with slight modifications. Briefly, sections were deparaffinised in xylene and rehydrated through an alcohol series. The material was then incubated in distilled water overnight, minced, digested in 2 ml of 0.5% pepsin (Sigma, St Louis, MO, USA) in 0.9% sodium chloride (pH 1.5) at 37°C for 30 min, sieved through a 37 μm mesh, and the pH neutralised. The sample was washed in phosphate-buffered saline (PBS) and the concentration adjusted to $10^6$ cells $ml^{-1}$. The cell suspension was then treated as follows: washed, incubated for 3 min on ice in PBS containing 0.1% Triton X-100 (Sigma), washed, incubated at 37°C for 20 min in PBS containing 180 units

RNAase $ml^{-1}$ (Sigma), washed and resuspended in PBS containing 0.3% polyethylene glycol (Research Products International, Mount Prospect, IL, USA) and 50 $\mu g\ ml^{-1}$ propidium iodide (Calbiochem, San Diego, CA, USA). All samples were examined microscopically for the presence of doublets, and if more than 5% were seen samples were syringed through a 21 gauge needle until the proportion of doublets was less than 5%. Additionally, doublets were electronically removed from analysis by gating on peak *vs* integral red fluorescence signals. All cell samples were analysed on the Coulter Electronics Profile II flow cytometer with PowerPak option (Coulter Electronics, Hialeah, FL, USA) at the Veterans Administration Lakeside Medical Center, collecting more than 50 000 events per sample. All samples were filtered through a 37 $\mu m$ nylon mesh just before analysis. Laser excitation was 15 mW at 488 nm and the standard optical filter configuration was used for red fluorescence light detection (488 nm dichroic, 457–502 nm long-pass laser blocking, 550 nm dichroic, 600 nm dichroic and 635 nm bandpass filters). Cell cycle analysis was done using the Modfit analysis routines (Verity Software House, Topsham, ME, USA), employing a single Gaussian function for each of the 2*n*, 4*n*, 8*n* etc. peaks, single-cut debri and rectangular S-phase functions. No population was classified as DNA aneuploid unless two distinct $G_0/G_1$ peaks were evident in the DNA content distribution (Hiddemann *et al.*, 1984). A DNA tetraploid population was defined as having a DNA index (DI) of 1.8–2.2 and a DNA octoploid population as having a DI of 3.6–4.4. A DNA tetraploid population was defined when the percentage of $G_2/M$, or tetraploid $G_0/G_1$ peak, was greater than 15% of the sum of [$\%G_0/G_1 + \%S + \%G_2/M$ (or DNA tetraploid peak)] (Rabinovitch, 1993). A DNA octaploid population was defined in an analogous manner. Since in these conventional DNA ploidy definitions, DNA ploidy class is defined by an artificial cut, abrupt changes in the percentage of a given DNA ploidy class can occur. For example, a sample with 16% 4*n* cells will be reported with both DNA diploid and tetraploid populations, whereas a sample with 14% 4*n* cells will be reported as 100% DNA diploid. Thus, the percentage of 2*n*, 4*n* and 8*n* cells are also reported. Since the DNA diploid or tetraploid $G_2/M$ population overlaps with the DNA tetraploid or octaploid $G_0/G_1$ respectively, proliferative fraction ($\%S + \%G_2/M$) data for each DNA ploidy population are not reported. However, no overlap of the S-phase fraction of each ploidy class occurs and, thus, the weight-averaged S-phase was calculated for each sample. Statistical comparisons of the percentage of DNA diploid and percentage of 2*n* fractions were done using the two-tailed Student's *t*-test.

### Results

Livers from all control animals and the 20 week ciprofibrate-fed animals appeared normal and did not contain any AHF, NN or HCC lesions. Examination of the livers of the 40 week ciprofibrate-fed animals revealed only AHF, but no nodules or HCC. These lesions were too small for separate isolation for flow cytometric analysis. The livers of all ten rats fed ciprofibrate for 20 months contained multiple grossly visible lesions. Histological examination of liver sections showed AHF, NN and HCC (Figures 1 and 2). The morphological features of these different lesions have been described previously (Rao *et al.*, 1990). Representative NNs, HCCs and NTC liver from each animal, based on H&E sections, were carefully selected for flow cytometry. A total of four NTCs, nine NNs and 27 HCCs were isolated.

The results for all animals fed control diet, regardless of age, and for the 20 week and 40 week ciprofibrate-fed animals was the same, showing either a predominant or exclusively DNA diploid population. The range in the per cent DNA diploid fraction for these samples was from 65–100% (data not shown).

Figure 3a and b shows typical results for an NTC and an NN sample, respectively, from an animal-fed ciprofibrate for 20 months. Prominent DNA tetraploid and DNA diploid populations are seen. All nine NNs had similar DNA ploidy patterns and the results are summarised in Table I. In no case were cell populations with a DI greater than 2.2 (>4c DNA content) seen, nor were any DNA aneuploid (as distinct from DNA heteroploid) populations observed. A statistically significant ($P = 0.007$) increase in the DNA diploid fraction was seen in NNs (mean = 50% ± 4%) as compared with non-tumorous adjacent areas (mean = 33% ± 6%). This difference is also reflected in the changes in average percentage of 2*n*, 4*n* and 8*n* cells with a statistically significant ($P = 0.03$) difference in percentage of 2*n* of NNs as compared with the NTCs.

Figure 4 shows three representative results for HCC specimens. A summary of HCC analyses are presented in Table I. In Figure 4a a sample with multiple heteroploid cell popula-

**Figure 1** Morphology of representative neoplastic nodules used for flow cytometric analysis. (H&E × 150).

**Figure 2** Morphology of representative hepatocellular carcinoma used for flow cytometric analysis (H&E × 110).

tions having DI of 1.0 (2c), 2.0 (4c), and 4.0 (8c) is shown. In no samples were significant populations having DI greater than 4.4 (8c) observed (see insert in Figure 4a). In Figure 4b, an HCC sample with four DNA ploidy populations (DI = 1.0, 1.8, 2.0 and 2.2) is shown. It should be noted that the results for all NN and NTC samples were consistent with DNA diploid or tetraploid populations. Seven of 27 HCC samples exhibited cell populations with DNA ploidy values (DI) distinct from those seen in NNs and NTCs. Additionally, five of seven of these HCC samples exhibited DNA aneuploid populations as distinct from DNA heteroploid (i.e consistent with 2c, 4c, 8c etc. DNA content). This is in contrast to the NN samples and NTC adjacent areas in which all populations were consistent with DNA heteroploid populations. Again, a statistically significant increase in the DNA diploid fraction ($P = 0.003$) and in the percentage of $2n$ ($P = 0.004$) as compared with surrounding NTC tissue was seen.

Evaluation of the weight-averaged per cent S-phase cells is shown in Table I. A trend to increasing average per cent S-phase was seen in the progression from NTCs to NNs to HCCs with statistically significant differences seen in the HCCs *vs* NTCs ($P = 0.003$) and HCCs *vs* NNs ($P = 0.01$) groups. Within the NN samples, three of nine had average per cent S-values greater than the NTC mean plus three standard deviations as compared with 16/24 in the HCC group.

### Discussion

In this study, DNA ploidy alterations were investigated in a rat model system of peroxisome proliferator-induced hepatocellular carcinoma. Examination of nine 'premalignant' NNs did not reveal the appearance of any new DNA ploidy classes as compared with surrounding non-tumorous controls (NTCs). Although, a significant increase in the fraction of DNA diploid or $2n$ DNA content cells was seen as compared with NTC. In examination of 27 hepatocellular carcinoma samples (HCC), seven exhibited the appearance of

**Figure 3** Flow cytometric distributions showing number of events *vs* red fluorescence (DNA content) for representative samples of non-tumorous surrounding tissue (**a**) and neoplastic nodule (**b**).

**Figure 4** Flow cytometric distributions showing number of events *vs* red fluorescence (DNA content) for representative HCC samples. Insert in (**a**) is HCC sample analysed at a lower amplifier gain setting to illustrate the lack of significant cells with DNA content greater than 8c.

**Table I** Summary of DNA ploidy and cell cycle data for NTC, NN and HCC samples

| *Sample* | *Average per cent S* | *Per cent diploid*[a] | *Per cent 2*n | *Per cent tetraploid*[a] | *Per cent 4*n | *Per cent octaploid*[a] | *Per cent 8*n |
|---|---|---|---|---|---|---|---|
| NTC ($n = 4$) | 2.5 ± 0.9 | 33 ± 6 | 33 ± 7 | 68 ± 6 | 65 ± 6 | – | 2 ± 1 |
| NN ($n = 9$) | 4.4 ± 3.7 | 50 ± 4 | 50 ± 4 | 50 ± 4 | 49 ± 5 | – | 1 ± 1 |
| HCC ($n = 27$) | 9.9 ± 9.1[b] | 52 ± 13 | 52 ± 14 | 46 ± 15 | 42 ± 17 | 3 ± 6 | 4 ± 5 |

[a] As defined in Materials and methods. [b] Only 24/27 HCC samples were adequate for S-phase determinations.

new DNA ploidy classes as compared with surrounding non-tumorous areas. The same increase in DNA diploid or 2*n* DNA content fraction as was seen in the NNs was observed in HCCs. A trend to increasing S-phase cells with progression from NTC to NN to HCC was seen with three of nine NN and 16/24 HCC samples having average per cent S greater than the NTC mean plus three standard deviations. Thus, these results do indicate an association of altered DNA ploidy and increased per cent S-phase with progression to HCC following treatment with a non-genotoxic peroxisome proliferator.

The predominance of a DNA diploid population in all control samples (20 weeks, 40 weeks or 20 months on control diet) and in the 20 and 40 week ciprofibrate-fed animals is in contrast with previous reports (Romagna and Zbinden, 1981; Schwarze *et al.*, 1984; Styles *et al.*, 1985; Styles *et al.*, 1988; Wang *et al.*, 1990) in which DNA tetraploid populations were predominant. In these previous reports, either image technology or cell isolation procedures that enriched the parenchymal cells were used. However, in this study, no enrichment of parenchymal cells was done. Thus, in these samples in which the entire tissue was analysed (no isolatable lesions were present), the results probably reflect the presence of non-parenchymal (diploid) cells within the liver. Interestingly, examination of the non-tumorous areas surrounding the NN and HCC lesions in the 20 month ciprofibrate-fed animals exhibited a more expected pattern with an average per cent DNA diploid fraction of 33%. When comparing the numbers from this study with others, it must be remembered that these results reflect nuclear ploidy and not cellular ploidy. Since this value is still somewhat on the high end of reported values for normal rat liver, inclusion of some non-parenchymal cells cannot be ruled out. Nonetheless, this is still a dramatic difference as compared with the normal control animals. Whether this reflects an effect of long-term ciprofibrate feeding of the animals or an effect of the NN or HCC on the surrounding normal tissue remains to be seen. Although less likely differences in the recovery of parenchymal *vs* non-parenchymal cells in controls *vs* ciprofibrate-fed animals cannot be ruled out. The preferential selection of population has been reported when using the Hedley method (Koss *et al.*, 1989).

In the isolation of NN and HCC, careful dissection was done to ensure that no normal tissue was included. Thus, the problems discussed above concerning parenchymal *vs* non-parenchymal cells does not apply. In both NNs and HCCs, a significant increase in the per cent DNA diploid fraction was seen as compared with NTC. It should be noted that even if inclusion of non-parenchymal cells in the NTC did occur the effect would be to mask rather than increase the differences seen in the NNs and HCCs. As such, this shift appears to be real and not an artifact of sample isolation and preparation. This result is similar to that seen in AHF or NN following treatment with a number of genotoxic agents (Schwarze *et al.*, 1984; Styles *et al.*, 1985; Sarafoff *et al.*, 1986; Denielsen *et al.*, 1988; Haesen *et al.*, 1988). Additionally, treatment with 2-acetylaminofluorene alone (Klose *et al.*, 1989) or *N*-nitrosomorpholine (Romagna and Zbinden, 1981) results in an increased diploid fraction. In contrast, studies with dimethylnitrosamine treatment have reported a shift to an increased tetraploid population (Digernes, 1983; Carlson and Abraham, 1985). Thus, although there may be a few exceptions, these results, as with the majority of studies employing genotoxic agents, would be consistent with a significant increase in the diploid fraction of cells being an early event in the development of HCC in rats following treatment with ciprofibrate. The average per cent S of the NNs, although higher, was not statistically different than the surrounding NTC.

The DNA ploidy alterations seen in the HCCs are similar to other reports (Stich, 1960; Nowell *et al.*, 1967; Becker *et al.*, 1971, 1973; Mori *et al.*, 1982; Digernes, 1983; Styles *et al.*, 1985; Denielsen *et al.*, 1988; Haesen *et al.*, 1988). However, the frequency of DNA aneuploidy in this model of HCC development is significantly lower (7/27) than has been reported in a number of studies using genotoxic agents (Stich, 1960; Nowell *et al.*, 1967; Becker *et al.*, 1971, 1973; Mori *et al.*, 1982). Although loss of DNA aneuploid populations has been seen with analysis of paraffin samples (Koss *et al.*, 1989), Digernes (1983) has also reported a low frequency of aneuploidy in HCC, and a high degree of variability, even among similar lesions in the same animal, has been reported (Denielsen *et al.*, 1988). The observation that progression to HCC was frequently accompanied by an increased per cent S-phase is not unexpected as increased proliferative fraction is a negative prognostic indicator in a number of malignancies (Bauer *et al.*, 1993). However, it is interesting to note that the increased frequency of lesions with higher per cent S and altered DNA ploidy occurred together in this model system. Correlation or association of increased per cent S in DNA aneuploid tumours or karyotypically unstable populations has been observed (Chen *et al.*, 1991; Waldman *et al.*, 1991; Steiner *et al.*, 1993), and it has been hypothesised that genetic changes may provide a proliferative advantage (Volpe, 1990) and that accumulation of alterations are associated with progression (Friend *et al.*, 1988). However, it should be noted that the low frequency of DNA aneuploid HCC in this model system does not imply that karyotypic or genetic instability is not still driving the process of progression to HCC (Sudilovsky *et al.*, 1991).

Only limited conclusions can be drawn about the underlying mechanisms of the genetic or DNA changes seen, since the analyses done in this study were at the gross DNA ploidy level, being insensitive to chromosomal changes involving fewer than several chromosomes. However, certainly in liver, a differentiated feature is the generation of tetraploid cells implying that with differentiation in liver there is a block in normal cytokinesis following cell replication, leading to the generation of DNA tetraploid and higher ploidy populations. With progression to malignancy there is a degree of loss of this differentiated feature or block and an increase in the diploid fraction of cells. This data would be consistent with this block in normal cytokinesis no longer functioning in the malignant cells. The appearance of non-integer DNA aneuploid populations does not occur until later in neoplastic progression when presumably, as with other tumour types, the loss of karyotype stability generates a wide range of both chromosomal structural and numerical changes. Elucidation of the primary mechanism (non-disjunction, etc.) underlying this instability awaits chromosomal studies.

DNA aneuploidy is common in human HCC and appears to be of prognostic value in patients (Popp and Marsman, 1991). The data examining DNA ploidy alterations in early lesions during the development of human HCC is limited, with reports of DNA aneuploidy being frequent in human AHF (Thomas *et al.*, 1992) and others reporting primarily diploid cells in AHF (Hoso and Nakanuma, 1991). However, the data suggest an earlier appearance of DNA aneuploidy in human HCC than in HCC in rats induced either by PP or genotoxic agents. It is mere speculation to hypothesise why this might be the case. Access to early lesions in the rat model system is far easier, and thus, even 'early' lesions in human may be further progressed than the early lesions that were examined in the rat. Clearly, the pattern of alterations in rat HCC varies with causative agent and differences in the primary causative agent might be related to the varying course of DNA alterations seen in human cells as well. Additionally, human cells are significantly more resistant to malignant transformation *in vitro* than rodent cells, particularly with regard to immortalisation (Shay *et al.*, 1989), and thus, greater genetic alteration, manifested as DNA aneuploidy, may occur sooner. However, it must be stated again that generation of DNA euploid malignant clones or foci is not inconsistent with underlying genetic instability (Sudilovsky *et al.*, 1991).

In conclusion, induction of HCC by the non-genotoxic peroxisome proliferator, ciprofibrate, in rat results in a significantly increased fraction of diploid cells in both NN and HCC as compared with surrounding non-tumorous tissue. As discussed above, this result is similar to results with a number of other hepatocarcinogens in the rat, and points to this being an important early event in the development of HCC in the rat. These early lesions did not exhibit a significantly increased per cent S-phase cells. However, careful assessment of the role of these changes in the earliest AHF or NN and comparison with the parenchymal cells of age-matched control animals or surrounding normal tissue will require alternative techniques such as image analysis. As has been generally found, HCC lesions exhibited an increased frequency of DNA ploidy alterations, a result consistent with continued genetic instability with progression to HCC. Additionally, a correlation of increased per cent S in the HCC lesions was also found.

**Acknowledgement**
This work was supported in part by the Veterans Administration Medical Service (Merit Award), the Fannie May Coleman Foundation and the National Cancer Institute.

### References

ARAKAWA M, KAGE M, SUGIHARA S, NAKASHIMA T, SUENAGA M AND OKUDA. (1986). Emergence of malignant lesions within an adenomatous hyperplastic nodule in a cirrhotic liver: Observations in five cases. *Gastroenterology*, **91**, 198–208.

BAUER KD, DUQUE RE AND SHANKEY TV. (1993). *Clinical Flow Cytometry Principles and Applications*. Williams and Wilkins: Philadelphia.

BECKER FF, FOX RA, KLEIN KM AND WOLMAN SR. (1971). Chromosome patterns in rat hepatocytes during N-2-fluorenylacetamide carcinogenesis. *J. Natl Cancer Inst.*, **46**, 1261–1269.

BECKER FF, KLEIN KM, WOLMAN SR, ASOFSKY R AND SELL S. (1973). Characterization of primary hepatocellular carcinomas and initial transplant generations. *Cancer Res.*, **33**, 3330–3338.

CARLSON J AND ABRAHAM R. (1985). Nuclear ploidy of neonatal rat livers: Effects of two hepatic carcinogens (Mirex and dimethylnitrosamine). *J. Toxicol. Environ. Health*, **15**, 551–559.

CHEN L-C, NEUBAUER A, KURISU W, WALDMAN FM, LJUNG B-M, GOODSON III W, GOLDMAN ES, MOORE II D, BALAZS M, MAYALL BH AND SMITH HS. (1991). Loss of heterozygosity on the short arm of chromosome 17 is associated with high proliferative capacity and DNA aneuploidy in primary breast cancer. *Proc. Natl Acad. Sci. USA*, **88**, 3847–3851.

CHO KR AND VOGELSTEIN B. (1992). Genetic alterations in the adenoma carcinoma sequence. *Cancer*, **70**, 1727–1731.

DENIELSEN HE, STEEN HB, LINDMO T AND REITH A. (1988). Ploidy distribution in experimental liver carcinogenesis in mice. *Carcinogenesis*, **9**, 59–63.

DIGERNES V. (1983). Chemical liver carcinogenesis: Monitoring of the process by flow cytometric DNA measurements. *Environ. Health Perspec.*, **50**, 195–200.

FARBER E AND CAMERON R. (1980). The sequential analysis of cancer development. *Adv. Cancer Res.*, **31**, 125–226.

FRIEND SH, DRYJA TP AND WEINBERG RA. (1988). Oncogenes and tumor-suppressing genes. *N. Engl. J. Med.*, **318**, 618–622.

GOLDSWORTHY TL, HANIGAN MH AND PITOT HC. (1986). Models of hepatocarcinogenesis in the rat-contrasts and comparisons. *CRC Crit. Rev. Toxicol.*, **17**, 61–89.

HAESEN S, DERIJCKE T, DELEENER A, CASTELAIN PH, ALEXANDRE H, PREAT V AND KIRSCH-VOLDERS M. (1988). The influence of phenobarbital and butylated hydroxytoluene on the ploidy rate in rat hepatocarcinogenesis. *Carcinogenesis*, **9**, 1755–1761.

HEDLEY DW, FRIEDLANDER ML, TAYLOR IW, RUGG CA AND MUSGROVE EA. (1983). Method for analysis of cellular DNA content of paraffin-embedded pathological material using flow cytometry. *J. Histochem. Cytochem.*, **31**, 1333–1335.

HIDDEMANN W, SCHUMANN J, ANDREEFF M, BARLOGIE B, HERMAN CJ, LEIF RC, MAYALL BH, MURPHY RF AND SANDBERG AA. (1984). Convention on nomenclature for DNA cytometry. *Cytometry*, **5**, 445–446.

HOSO M AND NAKANUMA Y. (1991). Cytophotometric DNA analysis of adenomatous hyperplasia in cirrhotic livers. *Virchows Archiv. A Pathol. Anat.*, **418**, 401–404.

KLOSE U, THIERAU D, GREIM H AND SCHWARZ LR. (1989). Centrifugal elutriation of hepatocytes from 2-acetylaminofluorene-treated rats and their characterization by flow cytometry. *Carcinogenesis*, **10**, 553–556.

KOSS LG, CZERNAK B, HERZ F AND WERSTO RP. (1989). Flow cytometric measurements of DNA and other cell components in human tumors: a critical appraisal. *Human Pathol.*, **20**, 528–548.

MARONPOT RR AND BOORMAN GA. (1982). Interpretation of rodent hepatocellular proliferative alterations and hepatocellular tumors in chemical safety assessment. *Toxicol. Pathol.*, **10**, 71–78.

MORI H, TANAKA T, SUGIE S, TAKAHASHI M AND WILLIAMS GM. (1982). DNA content of liver cell nuclei of *N*-2-fluorenylacetamide-induced altered foci and neoplasms in rats and human hyperplastic foci. *J. Natl Cancer Inst.*, **69**, 1277–1281.

NEWBERNE PM, SUPHAKARN V, PUNYARIT P AND CAMARGO JD. (1987). Nongenotoxic mouse liver carcinogens. In *Nongenotoxic Mechanisms in Carcinogenesis*. Butterworth BE and Slaga TJ (eds) pp. 165–178. Cold Spring Harbor Laboratory: New York.

NOWELL PC, MORRIS HP AND POTTER VR. (1967). Chromosomes of 'minimal deviation' hepatomas and some other transplantable rat tumors. *Cancer Res.*, **27**, 1565–1579.

PITOT HC AND SIRICA AE. (1980). The stages of initiation and promotion in hepatocarcinogenesis. *Biochim. Biophys. Acta*, **605**, 191–215.

PITOT HC, BARSNESS L, GOLDSWORTHY T AND KITAGAWA T. (1978). Biochemical characterization of stages of hepatocarcinogenesis after a single dose of diethylnitrosamine. *Nature (Lond.)*, **271**, 456–458.

POPP JA AND MARSMAN DS. (1991). Chemically induced cell proliferation in liver carcinogenesis. *Prog. Clin. Biol. Res.*, **369**, 389–395.

RABES HM. (1983). Development and growth of early preneoplastic lesions induced in the liver by chemical carcinogens. *J. Cancer Res. Clin. Oncol.*, **196**, 85–92.

RABINOVITCH PS. (1993). Practical considerations for DNA content and cell cycle analysis. In *Clinical Flow Cytometry Principles and Applications*, Bauer KD, Duque RE and Shankey TV (eds) pp. 117–142. Williams & Wilkins: Philadelphia.

RAO MS AND REDDY JK. (1991*a*). Toxicological implications of peroxisome proliferation. In *Hepatotoxicology*, Meeks R, Harrison S and Bull J (eds) pp. 621–646. CRC Press: Boca Raton, FL.

RAO MS AND REDDY JK. (1991*b*). An overview of peroxisome proliferator-induced hepatocarcinogenesis. *Environ. Health. Perspec.*, **93**, 205–209.

RAO MS, LALWANI ND AND REDDY JK. (1984). Sequential histologic study of rat liver during peroxisome proliferator [4-chloro-6-(2,3, xylidino)-2-pyrimidinylthio] acetic acid (WY-14,643)-induced carcinogenesis. *J. Natl Cancer Inst.*, **73**, 983–990.

RAO MS, TATEMATSU M, SUBBARAO V, ITO N AND REDDY JK. (1986). Analysis of peroxisome proliferator-induced preneoplastic and neoplastic lesions of rat liver for placental form of glutathione 2-transferase and gamma-glutamyl transpeptidase. *Cancer Res.*, **46**, 5287–5290.

RAO MS, YELDANDI AV, SUBBARO V AND REDDY JK. (1990). Evidence that ciprofibrate, a potent peroxisome proliferator, is a complete hepatocarcinogen in the rat. *Proc. Am. Assoc. Cancer Res.*, **31**, 86.

REDDY JK AND LALWANI ND. (1983). Carcinogenesis by hepatic peroxisome proliferators: Evaluation of the risk of hypolipidemic drugs and industrial plasticizers to humans. *CRC Crit. Rev. Toxicol.*, **12**, 1–53.

REDDY JK, RAO MS AND MOODY DE. (1976). Hepatocellular carcinomas in acatalasemic mice treated with nafenopin, a hypolipidemic peroxisome proliferator. *Cancer Res.*, **36**, 1211–1217.

ROMAGNA F AND ZBINDEN G. (1981). Distribution of nuclear size and DNA content in serial liver biopsies of rats treated with *N*-nitrosomorpholine, phenobarbital and butylated hydroxytoluene. *Exp. Cell Biol.*, **49**, 294–305.

SARAFOFF M, RABES HM AND DORMER P. (1986). Correlations between ploidy and initiation probability determined by DNA cytophotometry in individual altered hepatic foci. *Carcinogenesis*, **7**, 1191–1196.

SCHERER E. (1984). Neoplastic progression in experimental hepatocarcinogenesis. *Biochim. Biophys. Acta*, **738**, 219–236.

SCHWARZE PE, PETTERSEN EO, SHOAIB MC AND SEGLEN PO. (1984). Emergence of a population of small, diploid hepatocytes during hepatocarcinogenesis. *Carcinogenesis*, **5**, 1267–1275.

SHAY JW, WRIGHT WE AND WERBIN H. (1989). Defining the molecular mechanisms of human cell immortalization. *Biochim. Biophys. Acta*, **1072**, 1–7.

STEINER MG, HARLOW SP, COLOMBO E AND BAUER KD. (1993). Chromosomes 8, 12 and 17 copy number in Astler-Coller Stage C colon cancer in relation to proliferative activity and DNA ploidy. *Cancer Res.*, **53**, 681-686.

STICH HF. (1960). The DNA content of tumor cells. II. Alterations during the formation of hepatomas in rats. *J. Natl Cancer Inst.*, **24**, 1283-1297.

STYLES J, ELLIOTT BM, LEFEVRE PA, ROBINSON M, PRITCHARD N, HART D AND ASHBY J. (1985). Irreversible depression in the ratio of tetraploid:diploid liver nuclei in rats treated with 3'-methyl-4-dimethylaminoazobenzene (3'M). *Carcinogenesis*, **6**, 21-28.

STYLES JA, KELLY M, PRITCHARD NR AND ELCOMBE CR. (1988). A species comparison of acute hyperplasia induced by the peroxisome proliferator methylclofernapate: involvement of the binucleated hepatocyte. *Carcinogenesis*, **9**, 1647-1655.

SUDILOVSKY O, HINRICHSEN LI, HEI TK, WHITACRE CM, WANG JH, KASTURI S, JIANG SH, CECHNER R, MIRON S AND ABDUL-KARIM F. (1991). Genetic instability occurs sooner than expected: promotion, progression and clonality during hepatocarcinogenesis in the rat. In *Boundaries Between Promotion and Progression During Carcinogenesis*, Sudilovsky I (ed.) pp. 263-277. Plenum Press: New York.

TERADA T, HOSO M, SAITO K, KURUMAYA H AND NAKANUMA Y. (1989). Iron negative foci in siderotic macroregenerative nodules in human cirrhotic liver: A marker of incipient preneoplasia or neoplasia. *Arch. Pathol. Lab. Med.*, **113**, 916-920.

THOMAS RM, BERMAN JJ, YETTER RA, MOORE GW AND HUTCHINS GM. (1992). Liver cell dysplasia: A DNA aneuploid lesion with distinct morphologic features. *Hum. Pathol.*, **23**, 496-503.

VOLPE J. (1990). Genetic stability and instability in tumors. In *Molecular Biology of Cancer Genes*, Gluyser GM. (ed) pp. 9-23. Ellis Horwood: New York.

WALDMAN FM, CARROLL PR, KERSCHMANN R, COHEN MB, FIELD FG AND MAYALL BH. (1991). Centromeric copy number of chromosome 7 is strongly correlated with tumor grade and labeling index in human bladder cancer. *Cancer Res.*, **51**, 3807-3813.

WANG JH, HINRICHSEN LI, WHITACRE CM, CECHNER RL AND SUDILOVSKY O. (1990). Nuclear DNA content of altered hepatic foci in a rat liver carcinogenesis. *Cancer Res.*, **50**, 7571-7576.

WEISBURGER JH, YAMAMOTO RS, WILLIAMS GM, GRANTHAM PH, MATSUSHIMA T AND WEISBURGER EK. (1972). On the sulfate ester of *N*-hydroxy-*N*-2-fluorenylacetamide as a key ultimate hepatocarcinogen in the rat. *Cancer Res.*, **32**, 491-500.

WILLIAMS GM. (1980). The sequential analysis of liver cancer induction. *Biochim. Biophys. Acta*, **605**, 167-189.